# Supplementary material for: Joint analysis of expression levels and histological images identifies genes associated with tissue morphology
Source: Nat Commun. 2021 Mar 11;12:1609. doi: 10.1038/s41467-021-21727-x (PMC7952575; doi:10.1038/s41467-021-21727-x)
Supplement: Supplementary file 1 — Supplementary Information [file 41467_2021_21727_MOESM1_ESM.pdf]

Supplementary Information for: Joint analysis of gene expression and histological images identifies genes associated with tissue morphology

Supplementary Methods

CCA correlations rely on pairwise connections between tissue images and expression. We used permutations to validate that the correlations we observed between paired gene expression levels and image features did not occur by chance. To do this, we applied sparse CCA to the BRCA data after randomizing gene expression values in three ways. First, the samples were shuffled so that gene expression samples were paired with features from a random image. This type of permutation removes the correlation across image and gene expression observations, but retains the variation within each observation, including those due to biological and technical artifacts. Second, expression values were shuffled within each gene separately to remove dependency between genes. Third, values sampled from a normal distribution with mean zero and standard deviation one were used in place of the standardized gene expression values. The dot product of each corresponding pair of CCA variables is the metric of association that is maximized in CCA; this represents the empirical covariance of the normalized pair of observations. In the original data, this value for the first CCA component for the BRCA data is 5920. Using the three forms of randomized expression input data, this value is much lower, with mean values 3401 (std. dev. 105), 2,239 (std. dev. 502), and 1247 (std. dev. 23), respectively, for ten random replicates for each of the permutation types (Supplementary Fig 5), suggesting that CCA is indeed capturing meaningful correlations across the high-dimensional observations.

Supplementary Results

GTEx supervised ImageCCA. We trained the CAE end-to-end with a multilayer perceptron (MLP) using as class labels the tissue of origin of the sample. We removed 10% of the data to allow for testing. The generalization errors of this classifier were between $XXX\%$ and $XXX\%$ across tissues, where random assignment would be $XXX\%$ to $XXX\%$. We also performed ImageCCA with the image features estimated from this supervised CAE plus MLP. In the downstream image QTL analysis, we identified 28 image QTLs (FDR ≤ 0.1; Supplementary Table 11) in *heart–atrial appendage* samples.

Supplementary Tables

Supplementary Table 1. Unsupervised ImageCCA applied to the BRCA data. CCA_var is the CCA variable number (of 100); type is whether it was a gene or an image feature; name is the name of the feature; coefficient is the value of the CCA coefficient for that feature in that component. Only features with non-zero coefficients were included.

Supplementary Table 2. Supervised ImageCCA applied to the BRCA data. CCA_var is the CCA variable number (of 100); type is whether it was a gene or an image feature; name is the name of the feature; coefficient is the value of the CCA coefficient for that feature in that component. Only features with non-zero coefficients were included.

Supplementary Table 3. Gene Ontology analysis applied to results from unsupervised ImageCCA applied to the BRCA data. CCA var is the CCA component; ontology is one of BP, CC, or MF referring to the three different ontology types in GO; GO ID is the ID number of the GO term; term is the name of the GO term; annotated is the number of genes in that component; selected is the number of genes with that annotation; expected is the number of genes in that component that were expected to be associated with that GO term; the p-value is the uncorrected p-value of the enrichment of genes with that GO annotation in the component.

Supplementary Table 4. Unsupervised ImageCCA applied to the LGG data. CCA_var is the CCA variable number (of 100); type is whether it was a gene or an image feature; name is the name of the feature; coefficient is the value of the CCA coefficient for that feature in that component. Only features with non-zero coefficients were included.

Supplementary Table 5. Supervised ImageCCA applied to the LGG data. CCA_var is the CCA variable number (of 100); type is whether it was a gene or an image feature; name is the name of the feature; coefficient is the value of the CCA coefficient for that feature in that component. Only features with non-zero coefficients were included.

Supplementary Table 6. Gene Ontology analysis applied to results from unsupervised ImageCCA applied to the LGG data. CCA var is the CCA component; ontology is one of BP, CC, or MF referring to the three different ontology types in GO; GO ID is the ID number of the GO term; term is the name of the GO term; annotated is the number of genes in that component; selected is the number of genes with that annotation; expected is the number of genes in that component that were expected to be associated with that GO term; the p-value is the uncorrected p-value of the enrichment of genes with that GO annotation in the component.

Supplementary Table 7. Unsupervised ImageCCA applied to the GTEx data. CCA_var is the CCA variable number (of 100); type is whether it was a gene or an image feature; name is the name of the feature; coefficient is the value of the CCA coefficient for that feature in that component. Only features with non-zero coefficients were included.

Supplementary Table 8. Supervised ImageCCA applied to the GTEx data. CCA_var is the CCA variable number (of 100); type is whether it was a gene or an image feature; name is the name of the feature; coefficient is the value of the CCA coefficient for that feature in that component. Only features with non-zero coefficients were included.

Supplementary Table 9. Gene Ontology analysis applied to results from unsupervised ImageCCA applied to the GTEx data. CCA var is the CCA component; ontology is one of BP, CC, or MF referring to the three different ontology types in GO; GO ID is the ID number of the GO term; term is the name of the GO term; annotated is the number of genes in that component; selected is the number of genes with that annotation; expected is the number of genes in that component that were expected to be associated with that GO term; the p-value is the uncorrected p-value of the enrichment of genes with that GO annotation in the component.

Supplementary Table 10. Image morphology quantitative trait loci in the GTEx data in the unsupervised setting. SNP refers to the RSID of the genetic variant in the association; Gene refers to the gene for which the SNP was a cis-expression QTL; Image Feature refers to the number of the whitened image features in the association; P-Value refers to the uncorrected p-value of the association from linear regression; FDR refers to the Benjamini-Hochberg corrected false discovery rate (within tissue); Tissue refers to the tissue within which the test was performed.

Supplementary Table 11. Image morphology quantitative trait loci in the GTEx data in the supervised setting. SNP refers to the RSID of the genetic variant in the association; Gene refers to the gene for which the SNP was a cis-expression QTL; Image Feature refers to the number of the whitened image features in the association; P-Value refers to the uncorrected p-value of the association from linear regression; FDR refers to the Benjamini-Hochberg corrected false discovery rate (within tissue); Tissue refers to the tissue within which the test was performed.

Supplementary Figures

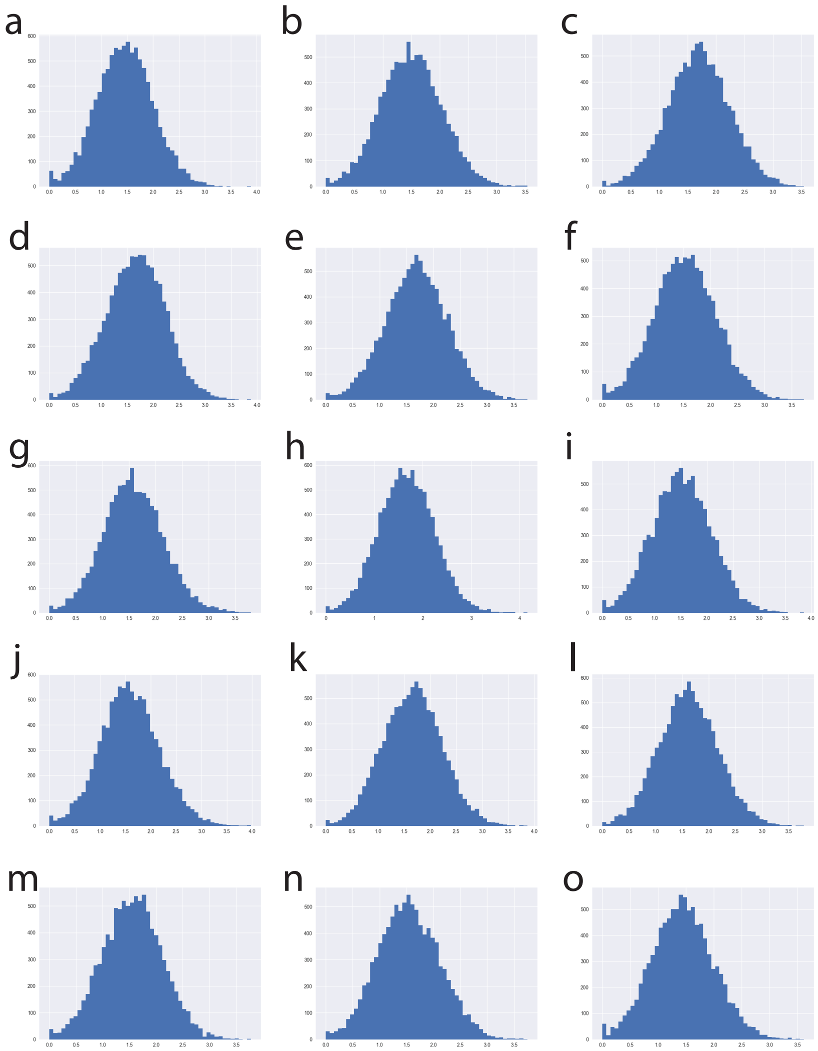

Supplementary Figure 1. Feature values tend to have a Gaussian distribution. Panels a-o show histograms of a feature value from each of the panels of a single image, with the feature values on the x-axis and the counts on the y-axis. This motivates taking the empirical mean across features; in the context of Gaussian-distributed features, this is equivalent to the empirical median.

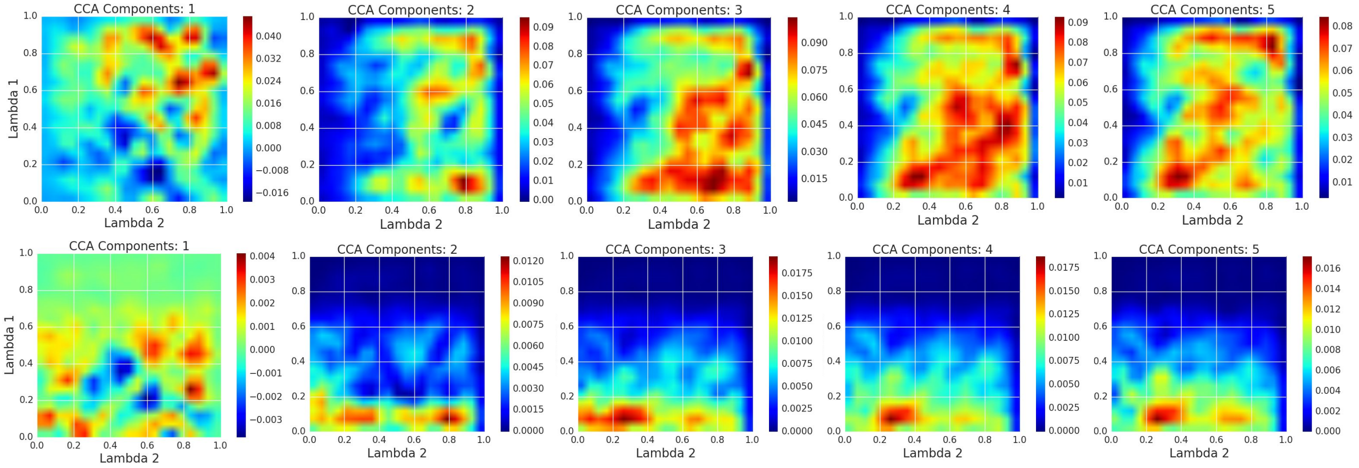

Supplementary Figure 2. Correlation between features and reconstructed features for LGG data. Each heatmap shows correlation as a function of λ_1, λ_2, and the number of CCA components. Low values of λ increase the amount of sparsity used in CCA. Correlations were computed using held out data, different from those used to fit CCA. Top row) Image features and reconstructions; Bottom row) gene expression features and reconstructions.

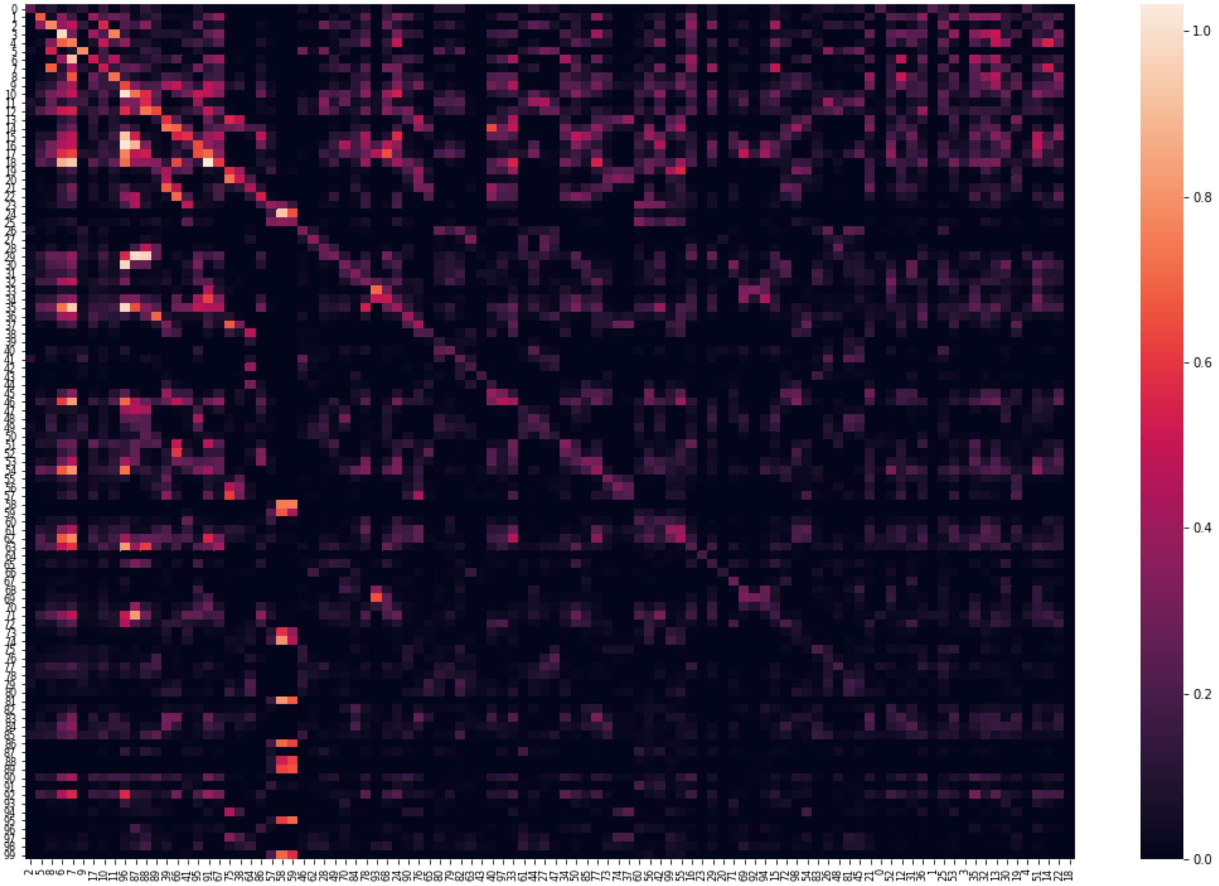

Supplementary Figure 3. Correlation between components with different hyperparameter settings. Heatmap shows the size of the intersection between the unique genes in a CCA component X and another CCA component X', divided by the minimum size of the set of the two CCA components. The axes are sorted to pair (along the diagonal) components that have overlapping gene sets. The $\lambda_2 = 0.1$ results are on the rows (these results are presented in the paper); the $\lambda_2 = 0.05$ results are on the columns (note permuted component numbers on columns).

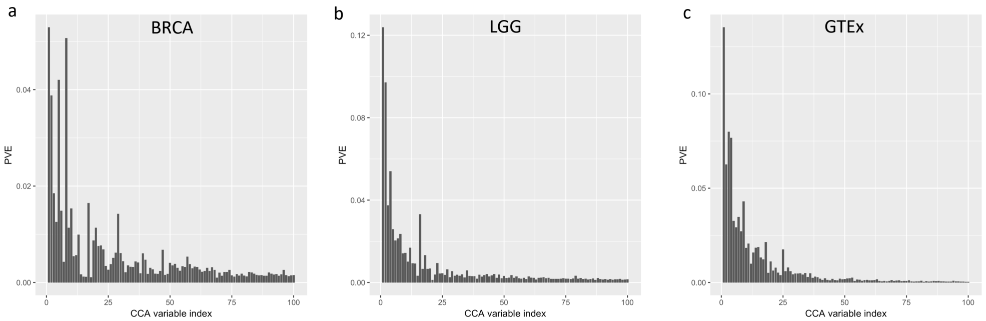

Supplementary Figure 4. Proportion of variance explained across components. Plots of the proportion of variance explained (PVE) for each of the 100 CCA components for the three data sets: a) BRCA; b) LGG; c) GTEx.

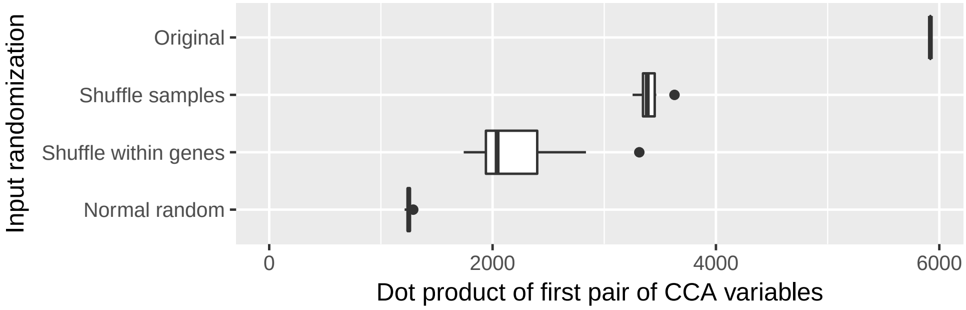

Supplementary Figure 5. Permuted observations dramatically weakens the sparse CCA correlations. The degree of dependence between the first pair of CCA variables, measured as the dot product, reflects the empirical covariance captured between CCA observations. We compare this to the dot product after permuting the data i) by shuffling sample labels of the gene expression samples, ii) by shuffling the expression values for each gene independently, and iii) by replacing standardized log-transformed expression values with numbers sampled from a standard normal distribution. The results of ten permutations using each of these methods are shown. The box hinges are the first quartile, the median, and third quartile of the image feature values, respectively. The lower whisker ranges from the bottom hinge to no less than 1.5 $\times$ IQR (inter-quartile range). The upper whisker ranges from the top hinge to no more than 1.5 $\times$ IQR. Outlier points defined as greater than or less than the whisker range.

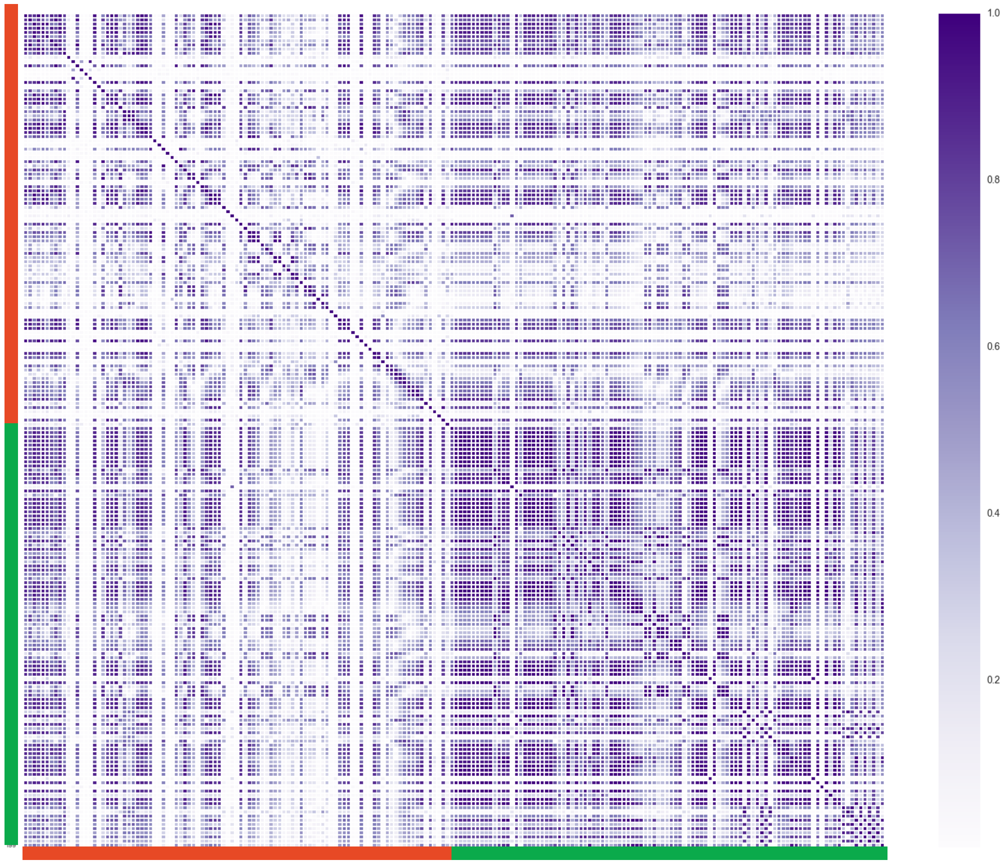

Supplementary Figure 6. Absolute value correlations between the components in ImageCCA and components in the supervised version of ImageCCA. Shade of dot represents the absolute value Pearson's correlation between all pairs of sparse factor loadings, with the legend on the left. The red labeled rows/columns represent components from ImageCCA; the green labeled rows/columns represent components from the supervised ImageCCA.

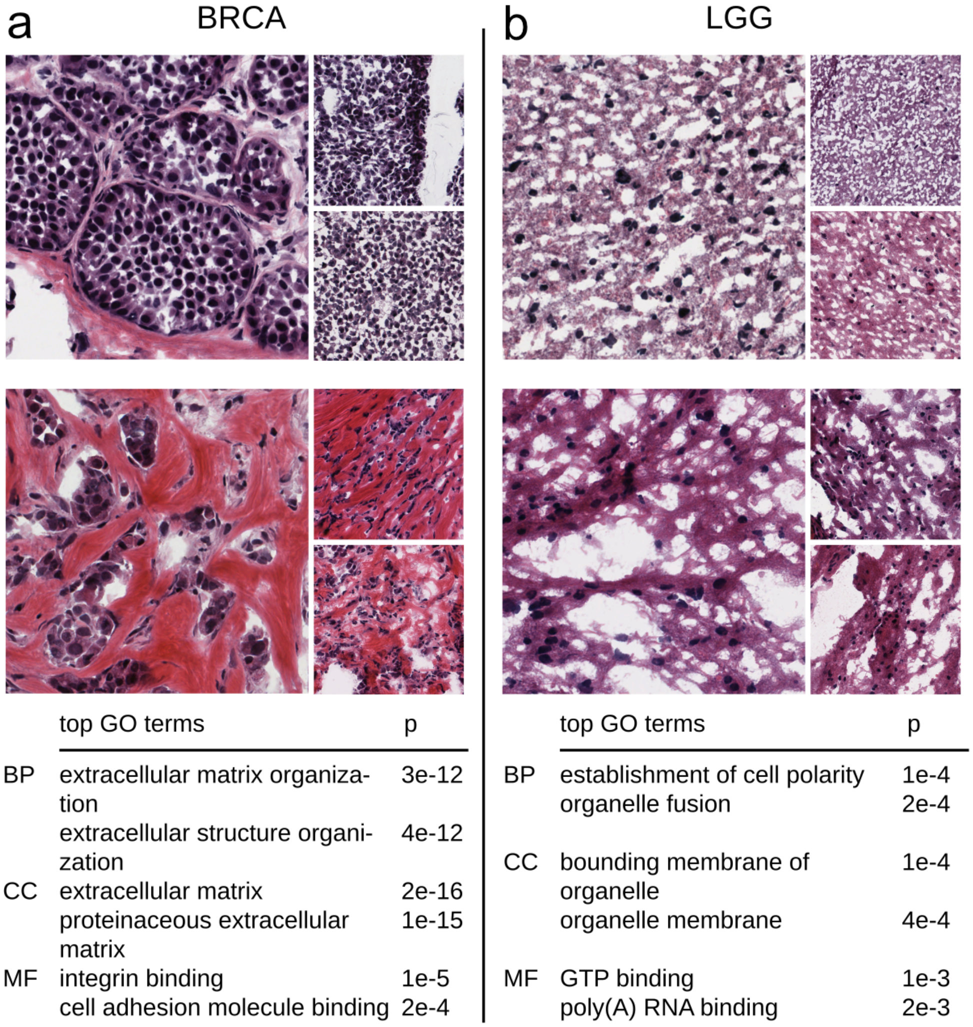

a BRCA

	top GO terms	p
BP	extracellular matrix organization	3e-12
	extracellular structure organization	4e-12
CC	extracellular matrix	2e-16
	proteinaceous extracellular matrix	1e-15
MF	integrin binding	1e-5
	cell adhesion molecule binding	2e-4

b LGG

	top GO terms	p
BP	establishment of cell polarity	1e-4
	organelle fusion	2e-4
CC	bounding membrane of organelle	1e-4
	organelle membrane	4e-4
MF	GTP binding	1e-3
	poly(A) RNA binding	2e-3

Supplementary Figure 7. Results using a CAE with an MLP for supervision to estimate an image embedding. We report images sampled from those with the most extreme (top and bottom 10%) loading values, and top two GO terms that are most enriched with the corresponding genes with extreme loading values in the same component. BP is *Biological Process*; CC is *Cellular Component*; MF is *Molecular Function*. The p-values reported are uncorrected Fisher's exact test. Panel a: the first component of the BRCA data; Panel b: the first component of the LGG data.

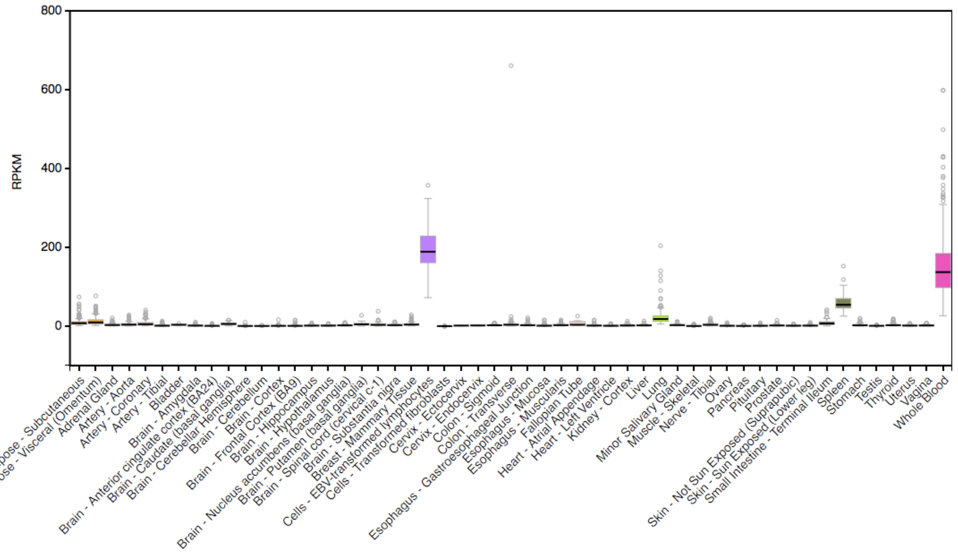

Supplementary Figure 8. Expression across tissues of the *PLEK* gene in GTEx tissue. Across the 44 tissues in GTEx, the *PLEK* gene is primarily expressed in LCLs and whole blood. The box hinges are the first quartile, the median, and third quartile of the image feature values, respectively. The lower whisker ranges from the bottom hinge to no less than 1.5 * IQR (inter-quartile range). The upper whisker ranges from the top hinge to no more than 1.5 * IQR. Outlier points defined as greater than or less than the whisker range.

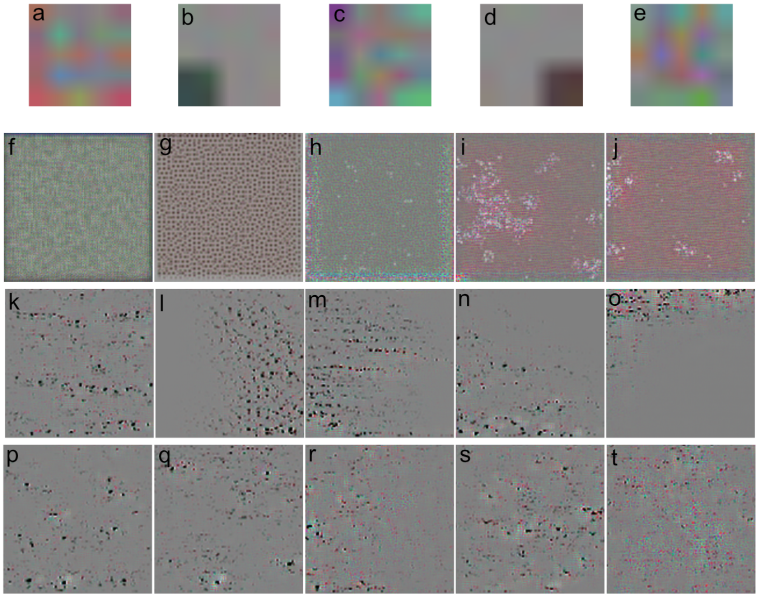

Supplementary Figure 9. Convolutional filters estimated from the CAE trained using GTEx images. a) through e) show convolutional filters estimated by the encoding portion of the CAE. f) through j) are the layer 1 filters, where we visualize the weights as a 5×5 image. k) through o) are the layer 2 filters, and p) through t) are the layer 3 filters. For layers 2 and 3, the filters were visualized by fixing a random image, then using gradient ascent, changing the image to get a better response from a fixed filter.

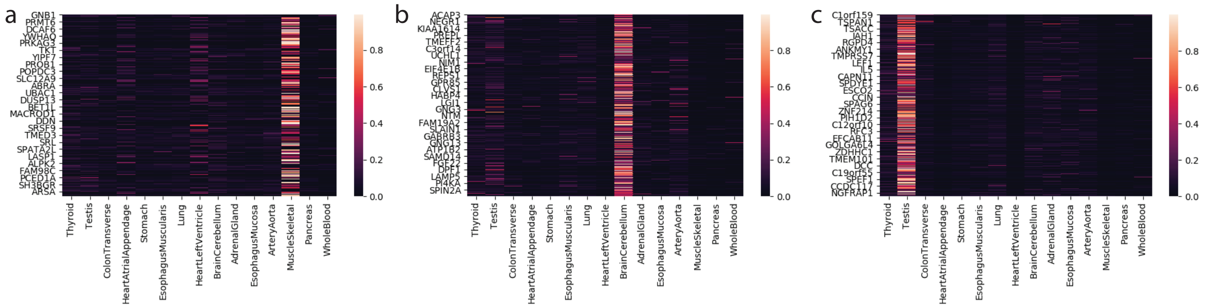

Supplementary Figure 10. Heatmaps showing the tissue specificity of randomly selected genes from three GTEx CCA components across different GTEx tissues a) component 1, showing enrichment in skeletal muscle; b) component 21, showing enrichment in cerebellum; c) component 24, showing enrichment in testis.

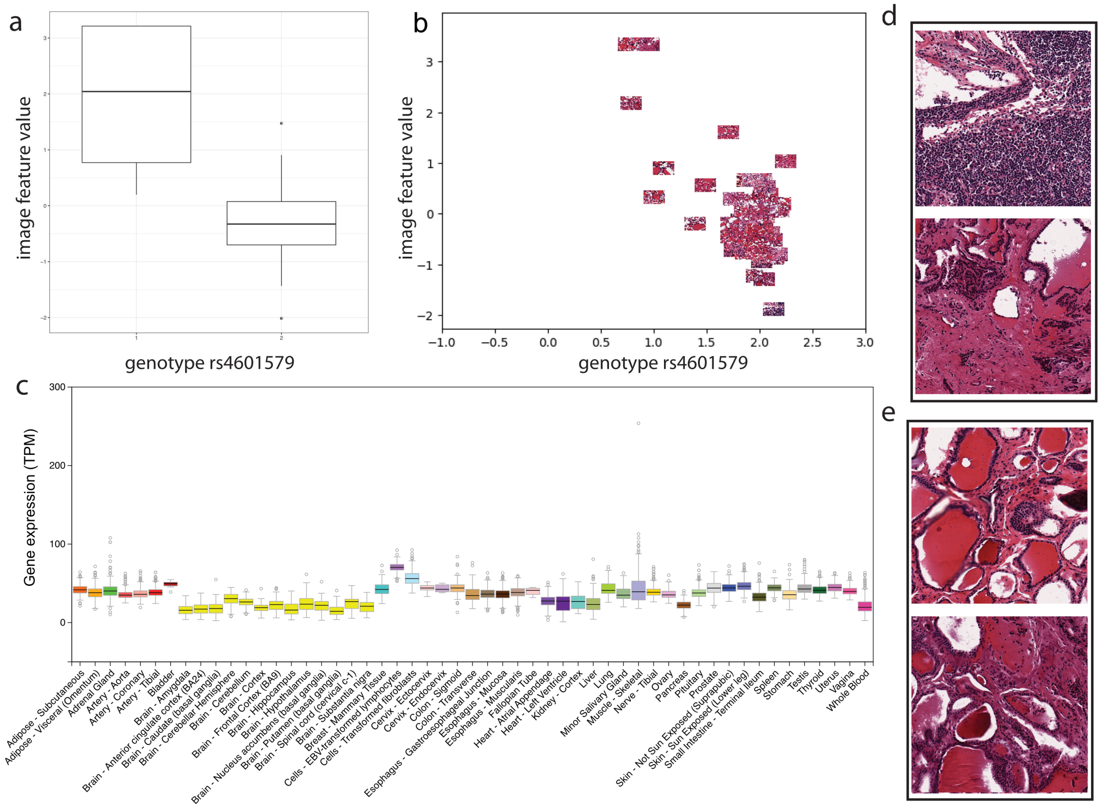

Supplementary Figure 11. Genotype and image feature association for an eQTL targeting death associated protein 3 (*DAP3*) in thyroid samples. *DAP3* and image feature 820 were identified jointly in CCA component 57. a) boxplot of association between genotype rs4601579 (x-axis) and image feature 820 values for all samples (y-axis), the box hinges are the first quartile, the median, and third quartile of the image feature values, respectively, the lower whisker ranges from the bottom hinge to no less than 1.5 * IQR (inter-quartile range), the upper whisker ranges from the top hinge to no more than 1.5 * IQR; b) same axes as a, but points are the thyroid images with jitter added to separate the images; c) relative abundance of *DAP3* expression across GTEx tissues, with *thyroid* showing substantial expression levels, boxplot defined the same as (a) with outlier points defined as greater than or less than the whisker range;; d) thyroid images in the top 10% of values for image feature 820; e) thyroid images in the bottom 10% of values for image feature 820.

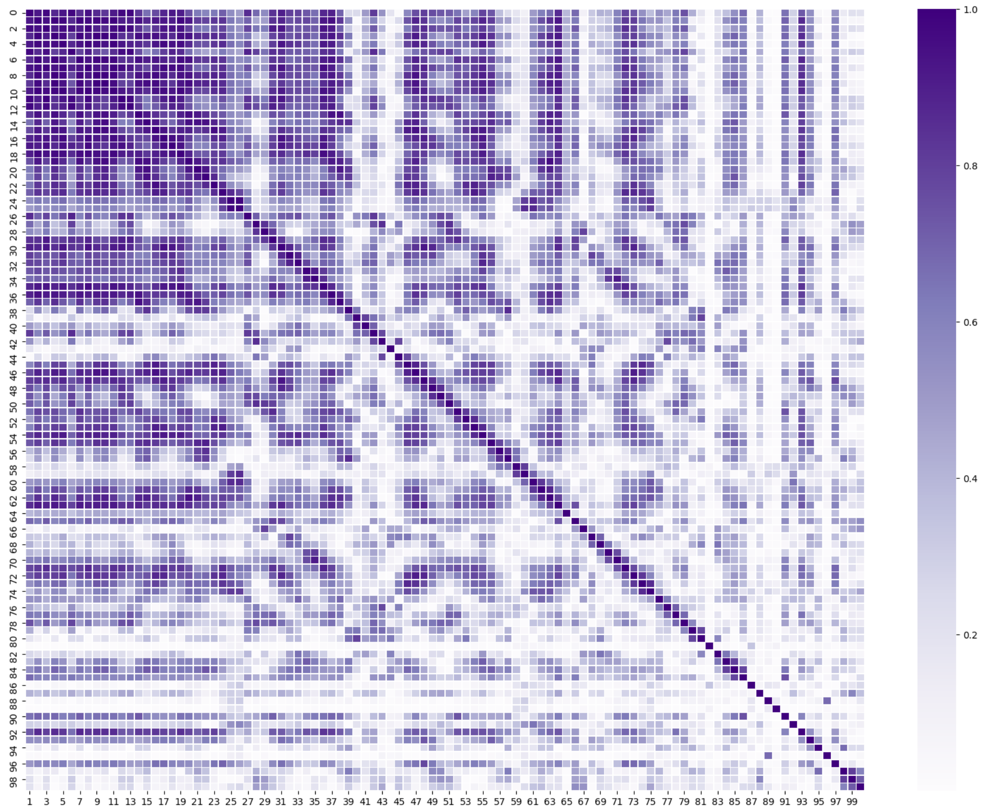

Supplementary Figure 12. Proportion of genes in the intersection of two components across 100 GTEx components. Values normalized by the size of the union of the number of genes in the two components. Despite their correlations, and similar correlation profiles with covariates, gene overlap across components shows that each component is fairly distinct in terms of the included genes and captures different sources of variation.
