# Supplementary material for: Joint analysis of expression levels and histological images identifies genes associated with tissue morphology
Source: Nat Commun. 2021 Mar 11;12:1609. doi: 10.1038/s41467-021-21727-x (PMC7952575; doi:10.1038/s41467-021-21727-x)
Supplement: Supplementary file 3 — Descriptions of Additional Supplementary Files [file 41467_2021_21727_MOESM3_ESM.pdf]

Descriptions of Additional Supplementary Files

Supplementary Data 1

Description: Unsupervised ImageCCA applied to the BRCA data. CCA var is the CCA variable number (of 100); type is whether it was a gene or an image feature; name is the name of the feature; coefficient is the value of the CCA coefficient for that feature in that component. Only features with non-zero coefficients were included.

Supplementary Data 2

Description: Supervised ImageCCA applied to the BRCA data. CCA var is the CCA variable number (of 100); type is whether it was a gene or an image feature; name is the name of the feature; coefficient is the value of the CCA coefficient for that feature in that component. Only features with non-zero coefficients were included.

Supplementary Data 3

Description: Gene Ontology analysis applied to results from unsupervised ImageCCA applied to the BRCA data. CCA var is the CCA component; ontology is one of BP, CC, or MF referring to the three different ontology types in GO; GO ID is the ID number of the GO term; term is the name of the GO term; annotated is the number of genes in that component; selected is the number of genes with that annotation; expected is the number of genes in that component that were expected to be associated with that GO term; the p-value is the uncorrected p-value of the enrichment of genes with that GO annotation in the component.

Supplementary Data 4

Description: Unsupervised ImageCCA applied to the LGG data. CCA var is the CCA variable number (of 100); type is whether it was a gene or an image feature; name is the name of the feature; coefficient is the value of the CCA coefficient for that feature in that component. Only features with non-zero coefficients were included.

Supplementary Data 5

Description: Supervised ImageCCA applied to the LGG data. CCA var is the CCA variable number (of 100); type is whether it was a gene or an image feature; name is the name of the feature; coefficient is the value of the CCA coefficient for that feature in that component. Only features with non-zero coefficients were included.

Supplementary Data 6

Description: Gene Ontology analysis applied to results from unsupervised ImageCCA applied to the LGG data. CCA var is the CCA component; ontology is one of BP, CC, or MF referring to the three different ontology types in GO; GO ID is the ID number of the GO term; term is the name of the GO term; annotated is the number of genes in that component; selected is the number of genes with that annotation; expected is the number of genes in that component that were expected to be associated with that GO term; the p-value is the uncorrected p-value of the enrichment of genes with that GO annotation in the component.

Supplementary Data 7

Description: Unsupervised ImageCCA applied to the GTEx data. CCA var is the CCA variable number (of 100); type is whether it was a gene or an image feature; name is the name of the feature; coefficient is the value of the CCA coefficient for that feature in that component. Only features with non-zero coefficients were included.

Supplementary Data 8

Description: Supervised ImageCCA applied to the GTEx data. CCA var is the CCA variable number (of 100); type is whether it was a gene or an image feature; name is the name of the feature; coefficient is the value of the CCA coefficient for that feature in that component. Only features with non-zero coefficients were included.

Supplementary Data 9

Description: Gene Ontology analysis applied to results from unsupervised ImageCCA applied to the GTEx data. CCA var is the CCA component; ontology is one of BP, CC, or MF referring to the three different ontology types in GO; GO ID is the ID number of the GO term; term is the name of the GO term; annotated is the number of genes in that component; selected is the number of genes with that annotation; expected is the number of genes in that component that were expected to be associated with that GO term; the p-value is the uncorrected p-value of the enrichment of genes with that GO annotation in the component.

Supplementary Data 10

Description: Image morphology quantitative trait loci in the GTEx data in the unsupervised setting. SNP refers to the RSID of the genetic variant in the association; Gene refers to the gene for which the SNP was a cis-expression QTL; Image Feature refers to the number of the whitened image features in the association; P-Value refers to the uncorrected p-value of the association from linear regression; FDR refers to the Benjamini-Hochberg corrected false discovery rate (within tissue); Tissue refers to the tissue within which the test was performed.

Supplementary Data 11

Description: Image morphology quantitative trait loci in the GTEx data in the supervised setting. SNP refers to the RSID of the genetic variant in the association; Gene refers to the gene for which the SNP was a cis-expression QTL; Image Feature refers to the number of the whitened image features in the association; P-Value refers to the uncorrected p-value of the association from linear regression; FDR refers to the Benjamini-Hochberg corrected false discovery rate (within tissue); Tissue refers to the tissue within which the test was performed
